# Distinct RGK GTPases Differentially Use α_1_- and Auxiliary β-Binding-Dependent Mechanisms to Inhibit Ca_V_1.2/Ca_V_2.2 Channels

**DOI:** 10.1371/journal.pone.0037079

**Published:** 2012-05-10

**Authors:** Tingting Yang, Akil Puckerin, Henry M. Colecraft

**Affiliations:** Department of Physiology and Cellular Biophysics, Columbia University, College of Physicians and Surgeons, New York, New York, United States of America; University of Waterloo, Canada

## Abstract

Ca_V_1/Ca_V_2 channels, comprised of pore-forming α_1_ and auxiliary (β,α_2_δ) subunits, control diverse biological responses in excitable cells. Molecules blocking Ca_V_1/Ca_V_2 channel currents (*I*_Ca_) profoundly regulate physiology and have many therapeutic applications. Rad/Rem/Rem2/Gem GTPases (RGKs) strongly inhibit Ca_V_1/Ca_V_2 channels. Understanding how RGKs block *I*_Ca_ is critical for insights into their physiological function, and may provide design principles for developing novel Ca_V_1/Ca_V_2 channel inhibitors. The RGK binding sites within Ca_V_1/Ca_V_2 channel complexes responsible for *I*_Ca_ inhibition are ambiguous, and it is unclear whether there are mechanistic differences among distinct RGKs. All RGKs bind β subunits, but it is unknown if and how this interaction contributes to *I*_Ca_ inhibition. We investigated the role of RGK/β interaction in Rem inhibition of recombinant Ca_V_1.2 channels, using a mutated β (β_2aTM_) selectively lacking RGK binding. Rem blocked β_2aTM_-reconstituted channels (74% inhibition) less potently than channels containing wild-type β_2a_ (96% inhibition), suggesting the prevalence of both β-binding-dependent and independent modes of inhibition. Two mechanistic signatures of Rem inhibition of Ca_V_1.2 channels (decreased channel surface density and open probability), but not a third (reduced maximal gating charge), depended on Rem binding to β. We identified a novel Rem binding site in Ca_V_1.2 α_1C_ N-terminus that mediated β-binding-independent inhibition. The Ca_V_2.2 α_1B_ subunit lacks the Rem binding site in the N-terminus and displays a solely β-binding-dependent form of channel inhibition. Finally, we discovered an unexpected functional dichotomy amongst distinct RGKs— while Rem and Rad use both β-binding-dependent and independent mechanisms, Gem and Rem2 use only a β-binding-dependent method to inhibit Ca_V_1.2 channels. The results provide new mechanistic perspectives, and reveal unexpected variations in determinants, underlying inhibition of Ca_V_1.2/Ca_V_2.2 channels by distinct RGK GTPases.

## Introduction

Ca^2+^ influx via high-voltage-activated Ca_V_1/Ca_V_2 Ca^2+^ channels links electrical signals to physiological responses in excitable cells, and consequently, regulates myriad biological functions ranging from muscle contraction to hormone and neurotransmitter release [1], [2]. Ca_V_1/Ca_V_2 channel activity is modulated by various intracellular signaling molecules, and this serves as a powerful method to alter physiology [1], [3]. Furthermore, molecules that selectively inhibit Ca_V_1/Ca_V_2 channels are current or prospective therapeutics for serious cardiovascular (e.g. hypertension, angina) and neurological (e.g. Parkinson's disease, neuropathic pain, stroke) diseases [4], [5], [6], [7], [8].

Rad/Rem/Rem2/Gem (RGK) proteins are a four-member subfamily of the Ras superfamily of monomeric GTPases [9], and are the most potent known intracellular inhibitors of Ca_V_1/Ca_V_2 channels [10], [11], [12]. RGK proteins are present in excitable tissue— including skeletal/cardiac muscle, nerve, and endocrine cells— suggesting that their inhibition of Ca_V_1/Ca_V_2 channels has physiological significance. Consistent with this notion, suppression of basal Rad expression in heart increases L-type Ca_V_1.2 calcium current (*I*_Ca,L_) and leads to cardiac hypertrophy [13], [14]. Mechanistically, RGK GTPases inhibit Ca_V_1/Ca_V_2 channels using multiple methods [15]. For example, Rem inhibits recombinant Ca_V_1.2 channels reconstituted in HEK 293 cells using at least three independent mechanisms [16]: (1) by decreasing the number of channels (*N*) at the cell surface; (2) by inhibiting open probability (*P*_o_) of surface channels; and (3) by partially immobilizing voltage sensors as reported by a reduced maximal gating charge (*Q*_max_).

A core unanswered question relates to the geographical localization of RGK binding site(s) on Ca_V_1/Ca_V_2 channel complexes responsible for *I*_Ca_ inhibition. Mature Ca_V_1/Ca_V_2 channels are macro-molecular complexes comprised minimally of a pore-forming α_1_ protein assembled with auxiliary β/α_2_δ subunits, and calmodulin [2], [17]. Ca_V_β is required for α_1_ trafficking to the plasma membrane, enhancing channel open probability (*P*_O_), and normalizing channel gating [18], [19]. All four RGKs bind Ca_V_βs and it has been widely assumed, though not proven, that the RGK/β interaction is essential for Ca_V_1/Ca_V_2 channel inhibition [10], [12], [15], [20]. This notion has been strongly challenged by a recent finding that β binding is not necessary for Gem inhibition of neuronal P/Q-type (Ca_V_2.1) channels [21]. This new provocative result raises several outstanding fundamental questions. First, it is now unclear whether the RGK/β interaction plays any role in *I*_Ca_ inhibition, or whether it is merely an unrelated epi-phenomenon. Second, though it has been proposed that RGKs may inhibit Ca_V_1/Ca_V_2 channels by binding directly to pore-forming α_1_ subunits [21], [22], to date no RGK binding site responsible for *I*_Ca_ reduction has been described for any α_1_-subunit isoform. Third, while it is formally possible that distinct RGKs may use different mechanisms and determinants to inhibit individual Ca_V_1/Ca_V_2 channels, this idea has not been explored.

Here, we report that Rem uses both β-binding-dependent and β-binding-independent mechanisms to inhibit recombinant Ca_V_1.2 channels. We identified a novel Rem binding region on the N-terminus of the pore-forming Ca_V_1.2 α_1C_ subunit that mediates β-binding-independent inhibition. The N-type (Ca_V_2.2) channel α_1B_ subunit lacks the Rem binding site in the N-terminus and displays only β-binding-dependent inhibition. Finally, we discovered that distinct RGK GTPases differ in their use of the two determinants for Ca_V_1.2 channel suppression— Rem and Rad use both β-binding-dependent and independent mechanisms, whereas Gem and Rem2 solely utilize a β-binding-dependent mode of inhibition.

## Results

### Rem inhibits Ca_V_1.2 channels using both β-binding-dependent and β-binding-independent mechanisms

**Figure 1:**
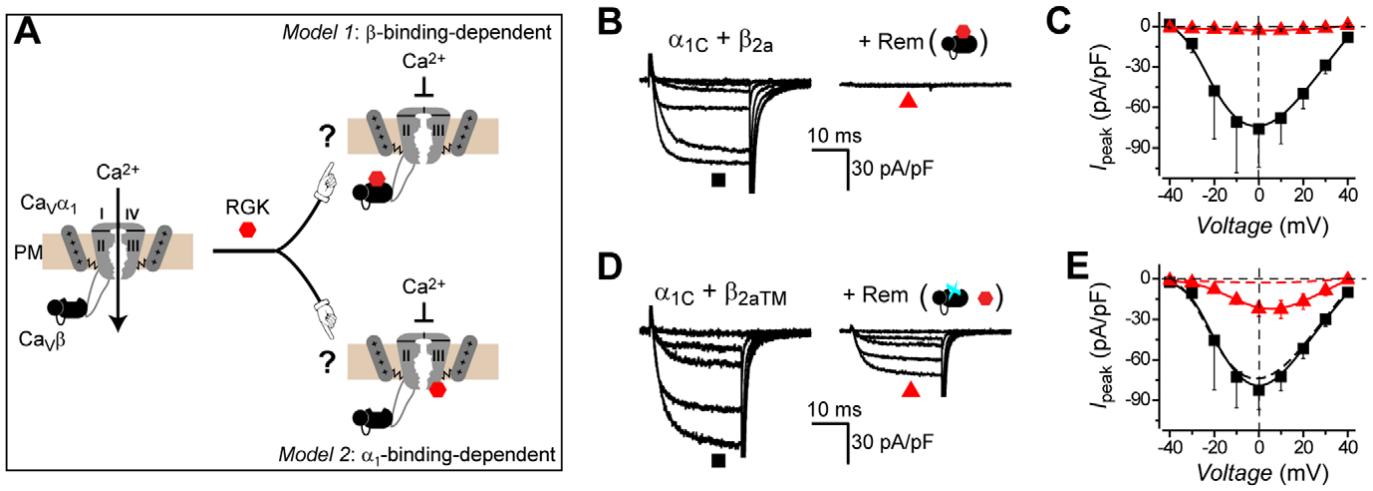
Rem inhibits Ca_V_1.2 channels using both β-binding-dependent and independent mechanisms. (A) Alternative models for Rem functional interaction with Ca_V_1.2 channel complex. (B) Exemplar Ba^2+^ currents from HEK 293 cells expressing wild-type Ca_V_1.2 (α_1C_+β_2a_) in the absence (*left*) or presence (*right*) of Rem. (C) Population current density (*I*_peak_) vs. voltage relationships for wild-type Ca_V_1.2 channels in the absence (▪, *n* = 6 for each point) or presence (red ▴, *n* = 5 for each point) of Rem. Data are means ± S.E.M. (D, E) Data for mutant Ca_V_1.2 channels (α_1C_+β_2aTM_) in the absence (▪, *n* = 8 for each point) or presence (red ▴, *n* = 10 for each point) of Rem. Same format as B, C. In *E*, data from wild-type Ca_V_1.2 channels are reproduced (dotted lines) to facilitate direct visual comparison.

Rem potently inhibits recombinant Ca_V_1.2 channels (α_1C_/β_2a_) reconstituted in HEK 293 cells (Fig. 1 B and C). Cells transiently transfected with α_1C_+β_2a_ generate robust *I*_Ca,L_ which is virtually eliminated (96% inhibition) when Rem is co-expressed (Fig. 1 B and C). It is unknown whether this dramatic effect is mediated through Rem binding to the auxiliary β, the pore-forming α_1C_ subunit, or both (Fig. 1A). To address this issue, we introduced three point mutations (D243A, D319A and D321A) into β_2a_ to generate a mutant (β_2aTM_) that selectively loses binding to RGK proteins, as previously demonstrated [23] and confirmed here (Fig. S1). Cells expressing mutant Ca_V_1.2 channels reconstituted with α_1C_+β_2aTM_ yielded strong *I*_Ca,L_ with amplitude and voltage-dependence indistinguishable from wild-type Ca_V_1.2 (Fig. 1 D and E), demonstrating that the mutations did not adversely affect the structure and functional interaction of β with α_1C_. Rem inhibited *I*_Ca,L_ through mutant α_1C_+β_2aTM_ Ca_V_1.2 channels (Fig. 1 D and E). However, the magnitude of Rem inhibition of mutant channels (74%) was significantly less than observed with wild type Ca_V_1.2 (Fig. 1). The intermediate impact of Rem on α_1C_+β_2aTM_ channels indicates Rem inhibits Ca_V_1.2 channels using both β-binding-dependent and independent mechanisms.

**Figure 2:**
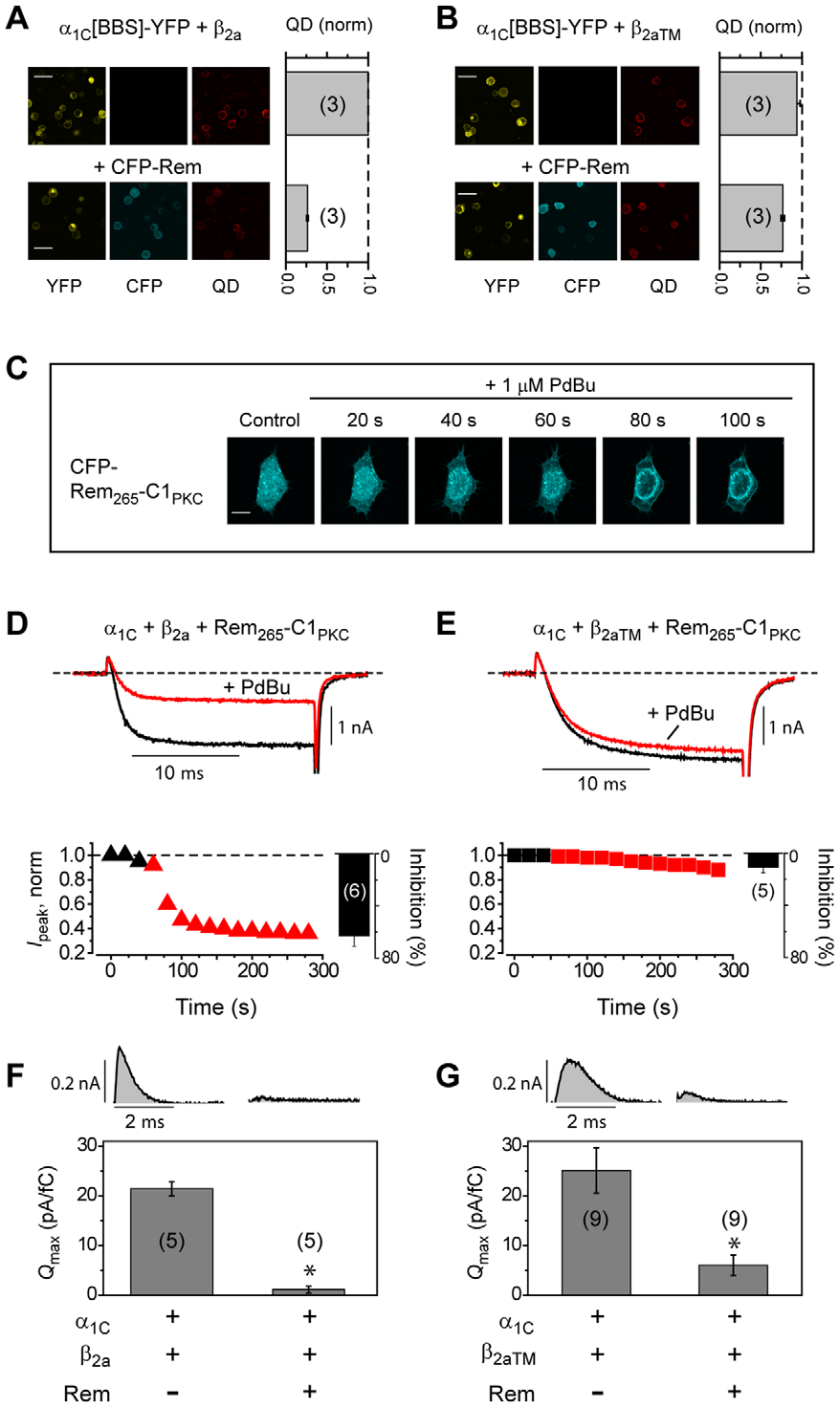
Distinct mechanisms of Rem inhibition of Ca_V_1.2 differentially depend on Rem/β interaction. (A, B) Differential impact of CFP-Rem on surface density of wild-type (α_1C_[BBS]-YFP+β_2a_) and mutant (α_1C_[BBS]-YFP+β_2aTM_) Ca_V_1.2 channels, respectively, using a surface channel quantum dot labeling method. Confocal images for corresponding imaging channels were obtained with identical instrument settings. Scale bar, 25 µm. (C) Rapid recruitment of CFP-Rem_265_-C1_PKC_ to the plasma membrane induced by 1 µM PdBu. Scale bar, 8 µm. (D, E) PdBu-induced membrane translocation of CFP-Rem_265_-C1_PKC_ concomitantly inhibits wild-type (α_1C_+β_2a_), but not mutant (α_1C_+β_2aTM_) Ca_V_1.2 channels. (F, G) Rem inhibits gating currents and *Q*_max_ in both wild-type and mutant Ca_V_1.2 channels. * *P*<0.05 when compared to the corresponding without Rem data using Student's two-tailed unpaired *t* test.

We previously reported that Rem inhibits Ca_V_1.2 channels using multiple, independent methods: decreasing *N*, *P*_o_, and *Q*_max_ [16]. We investigated which, if any, of these distinct mechanisms is dependent on Rem binding to β. To quantitatively determine the relative Ca_V_1.2 surface density we introduced a 13-residue high-affinity bungarotoxin (BTX) binding site (BBS) into the extracellular domain II S5–S6 loop in α_1C_-YFP [16]. Surface α_1C_[BBS]-YFP was detected in non-permeabilized cells by sequential exposure to biotinylated BTX and streptavidin-conjugated quantum dot (QD). Labeled cells are then subject to flow cytometry, permitting high throughput measurements of fluorescence signals [16], [24] (Fig. S2). Cells expressing α_1C_[BBS]-YFP+β_2a_ displayed a strong QD_655_ fluorescence signal (Fig. 2A, *top row*), indicating an abundance of channels at the cell surface. Co-expression of CFP-Rem with wild-type Ca_V_1.2 markedly decreased *N*, as reported by a ∼75% decrease in mean QD_655_ fluorescence (Fig. 2A; normalized mean QD_655_ fluorescence = 0.26±0.01, *n* = 3 independent flow cytometry experiments in cells co-expressing CFP-Rem compared to control cells expressing α_1C_[BBS]-YFP+β_2a_ alone). These results are consistent with our previous observations [16]. Cells expressing α_1C_[BBS]-YFP+β_2aTM_ displayed a similar channel surface density as control α_1C_[BBS]-YFP+β_2a_ cells (Fig. 2B; normalized mean QD_655_ fluorescence = 0.94±0.04, *n* = 3). Interestingly, CFP-Rem barely decreased QD_655_ fluorescence in cells expressing α_1C_[BBS]-YFP+β_2aTM_ (Fig. 2B; normalized mean QD_655_ fluorescence = 0.77±0.02, *n* = 3), compared to the substantial drop observed with control channels (Fig. 2A). Therefore, the ability of Rem to reduce *N* is critically dependent on its capacity to bind β.

A second mode of Rem inhibition of Ca_V_1.2 involves a reduction in channel *P*_o_ that depends on membrane targeting of Rem's nucleotide binding domain (NBD) [16], [20]. When expressed in cells, wild-type Rem autonomously targets to the inner leaflet of the plasma membrane via electrostatic and hydrophobic interactions afforded by basic and aromatic residues in the distal C-terminus [25]. A Rem truncation mutant, Rem_265_, featuring a deletion of the final 32 amino acid residues in the C-terminus, loses both membrane targeting and the ability to block *I*_Ca_ [12], [16], [20]. Replacing the deleted 32 residues with a generic membrane-targeting domain rescues the capacity to inhibit *I*_Ca_ [26]. We exploited this feature to generate an inducible Ca_V_ channel inhibitor by placing the C1 domain of protein kinase Cγ (PKCγ) to the end of CFP-Rem_265_ [16]. When expressed in cells, the resulting construct, CFP-Rem_265_-C1_PKC_, is cytosolic but can be rapidly recruited to the plasma membrane with the phorbol ester, PdBu (Fig. 2C). In α_1C_+β_2a_ channels, membrane recruitment of Rem_265_-C1_PKC_ results in an attendant rapid and substantive 60% decrease in *I*_Ca_ (Fig. 2D), which is solely due to a decrease in *P*_o_ [16], [20]. In sharp contrast, α_1C_+β_2aTM_ channels were unaffected by membrane-recruitment of Rem_265_-C1_PKC_ (Fig. 2E). The slight 10% reduction in *I*_Ca_ observed in this group is commensurate with the normal amount of channel rundown observed in these time course experiments. These results establish that this Rem-induced reduced-*P*_o_ mechanism of channel inhibition is also mediated through the Rem/β interaction.

A third characteristic functional impact of Rem on Ca_V_1.2 channels is a reduction of *Q*_max_ that occurs even when the decrease in *N* is accounted for, and is likely accomplished by a Rem-induced partial immobilization of α_1C_ voltage sensors [16]. Wild-type α_1C_+β_2a_ channels yield large ON gating currents and *Q*_max_, which are almost eliminated in the presence of CFP-Rem (Fig. 2F). Qualitatively similar results were obtained with mutant α_1C_+β_2aTM_ channels, which displayed a large *Q*_max_ that was significantly reduced by CFP-Rem (Fig. 2G). Therefore, unlike the effects on *N* and *P*_o_, binding to β is not necessary for Rem-induced decrease of Ca_V_1.2 *Q*_max_.

### Identification of a novel Rem binding region on the pore-forming α_1C_ subunit

**Figure 3:**
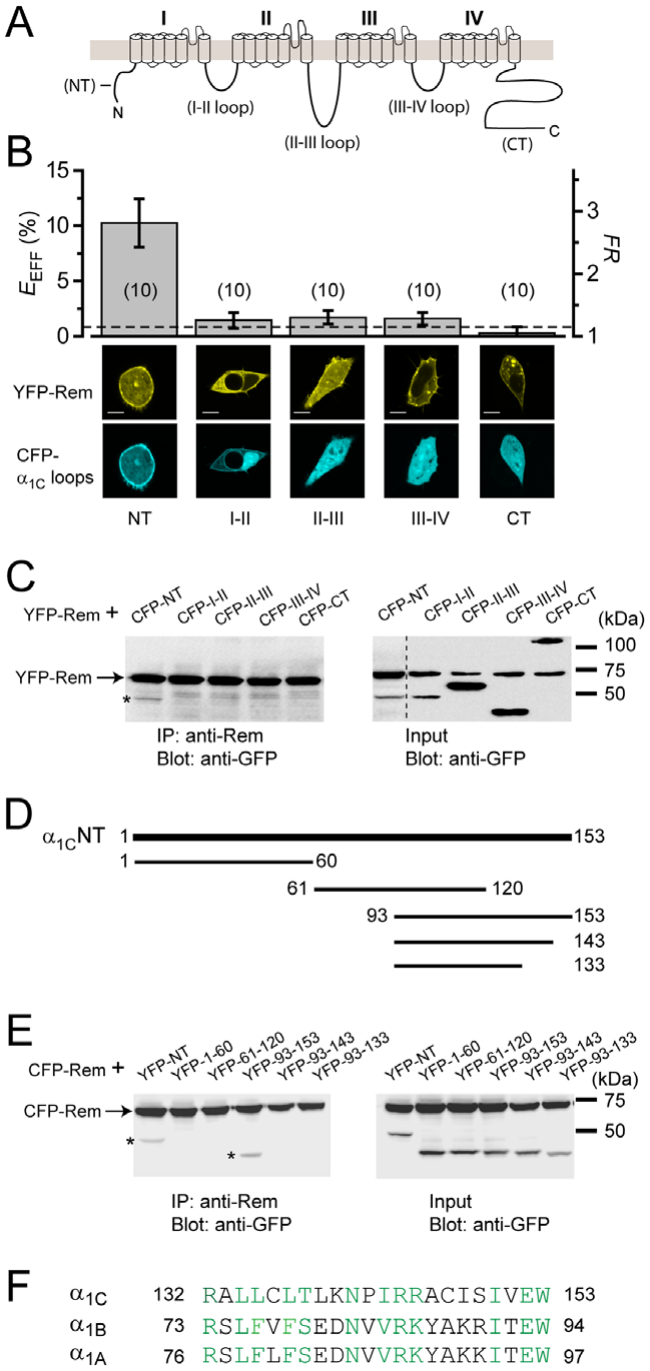
Rem binds α_1C_ N-terminus. (A) Schematic of α_1C_ showing four homologous transmembrane domains (I–IV), intracellular N/C termini and domain-connecting loops. (B) *Top*, interaction of individual CFP-tagged α_1C_ intracellular loops and termini with YFP-Rem probed using FRET. Dotted line represents YFP-Rem+CFP (*n* = 10). *Bottom*, confocal images. Scale bar, 8 µm. (C) CFP-tagged α_1C_NT co-immunoprecipitates with YFP-Rem. All the co-ip lanes and the first input lane were from the same gel. The rest of the input lanes were from a second gel run simultaneously because there were insufficient lanes available in the first gel to accommodate all samples, including marker lanes. Hence, in the input gel image (*right*) the first lane (CFP-NT) was spliced onto the rest of the lanes (dotted line). The co-ip gels have been cropped to remove light chain IgG bands from the precipitating antibody. (D) Schematic of α_1C_NT peptide fragments. (E) Co-immunoprecipitation of YFP-tagged α_1C_NT peptide fragments with CFP-Rem. (F) Sequence comparison of last 22 N-terminus residues among distinct Ca_V_1/Ca_V_2 channel α_1_ subunits.

The most parsimonious explanation for the existence of a β-binding-independent mode of Rem-induced block of *I*_Ca,L_ is that Rem directly binds α_1C_ to initiate this form of Ca_V_1.2 inhibition. However, to date, no such functional Rem binding site on α_1C_ has been described. Given that Rem is localized to the intracellular side of the plasma membrane, we hypothesized the existence of a Rem binding site somewhere within the major cytoplasmic regions (N-terminus, I–II loop, II–III loop, III–IV loop, and C-terminus) of α_1C_ (Fig. 3A). We searched for such a binding site using two complementary methods. First, we used fluorescence resonance energy transfer (FRET) to probe for an interaction between YFP-Rem and CFP-tagged intracellular domains of α_1C_ (Fig. 3B). Using a three-cube FRET method [27], [28], we found that only CFP-tagged α_1C_ N-terminus (CFP-α_1C_NT) yielded an appreciable FRET signal when co-expressed with YFP-Rem (Fig. 3B). None of the other CFP-tagged α_1C_ intracellular loops yielded a FRET signal significantly above control cells expressing YFP-Rem+CFP (Fig. 3B, dotted line). The FRET results were not due to differences in the stoichiometry of donor to acceptor molecules since the estimated ratio of donor (*N*_D_) to acceptor (*N*_A_) molecules [27], [28] was similar among the different groups (Fig. S3). The FRET results aligned with visual evidence of protein co-localization (Fig. 3). When expressed individually, YFP-Rem is enriched at the plasma membrane whereas CFP-α_1C_NT has a mostly diffuse fluorescence through the cytosol and in the nucleus (Fig. S4). However, when co-expressed with YFP-Rem, a fraction of the CFP-α_1C_NT present in cells was targeted to the plasma membrane, tracking the membrane localization of Rem and providing visual evidence of an interaction (Fig. 3B; Fig. S4).

As a complementary approach, we used co-immunoprecipitation (co-IP) assays to determine interaction between YFP-Rem and individual CFP-tagged α_1C_ intracellular domains co-transfected into HEK 293 cells (Fig. 3C). All CFP-tagged α_1C_ intracellular domains and YFP-Rem were well expressed (Fig. 3C, *input*). Only CFP-α_1C_NT co-IPed with YFP-Rem (Fig. 3C), corroborating the results from FRET and protein co-localization approaches (Fig. 3B). As a further control experiment, we observed no pull down of CFP-α_1C_NT with anti-Rem antibody in cells transfected with CFP-α_1C_NT alone (*i.e.,* no YFP-Rem co-expressed; not shown). We were surprised to find no binding between Rem and α_1C_ C-terminus (α_1C_CT) given a recent report that these two proteins interact [29]. The reasons for this disparity are unclear. However, the fact that using three independent approaches (FRET, co-localization analyses, and co-IP) we could observe no interaction between Rem and α_1C_CT while detecting association with α_1C_NT effectively rules out the potential trivial explanation of a false negative result that could conceivably be obtained with any one method. One possibility is that the presence of fluorescent protein tags on Rem and α_1C_CT may occlude or weaken this interaction to a point where it is undetectable in our different assay conditions.

**Figure 4:**
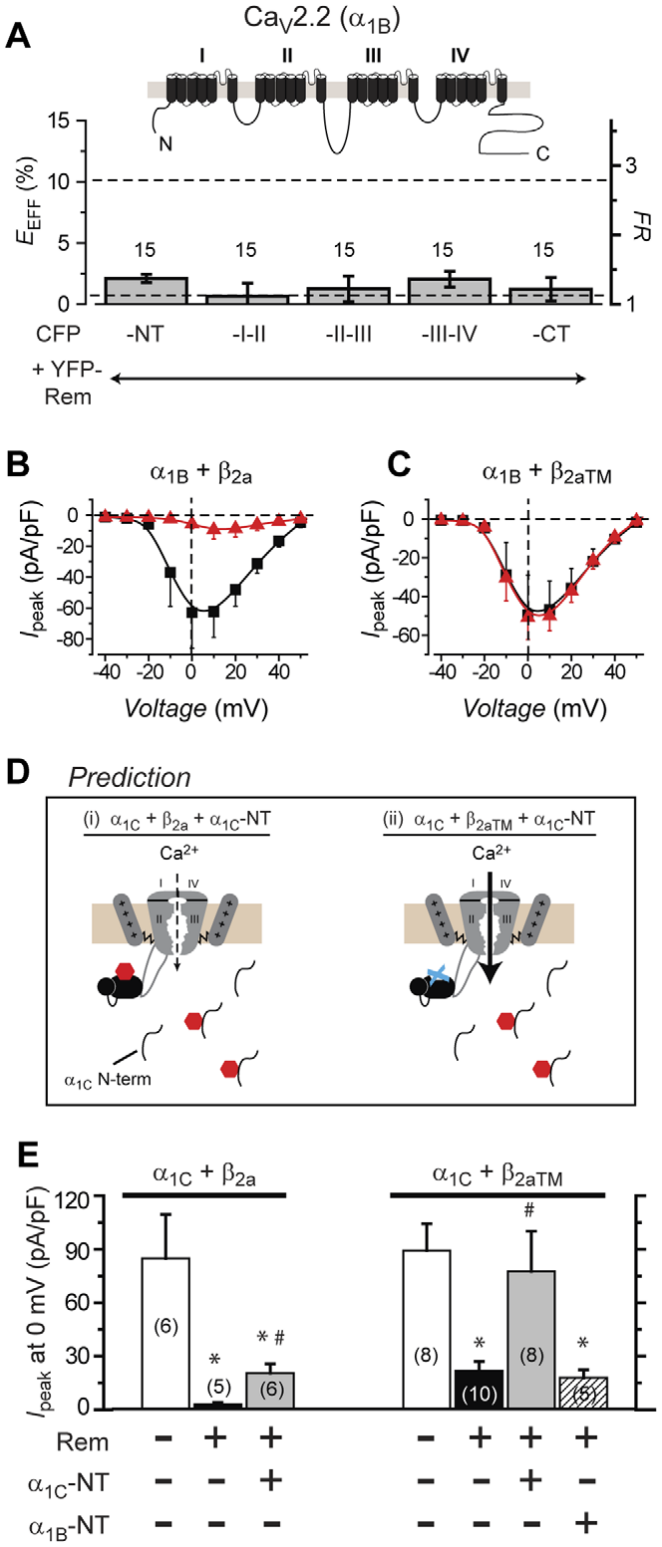
Rem interaction with α_1C_ N-terminus mediates β-binding-independent inhibition. (A) *Top*, topography of Ca_V_2.2 α_1B_ subunit. *Bottom*, interaction of Ca_V_2.2 α_1B_ intracellular domains with YFP-Rem probed using FRET. Dotted lines represent FRET data from YFP-Rem+CFP-α_1C_NT and YFP-Rem+CFP, respectively. (B, C) Population *I*_peak_-*V* relationships for wild type (α_1B_+β_2a_) and mutant (α_1B_+β_2aTM_) Ca_V_2.2 channels, respectively, in the absence (▪, *n* = 5 for wild type channels, and *n* = 9 for mutant channels) or presence (red ▴, *n* = 5 for wild type channels, and *n* = 10 for mutant channels) of Rem. Data are means ± S.E.M. (D) Schematic showing rationale and predictions for α_1C_ N-terminus over-expression experiments. (E) Histogram showing impact of α_1C_ or α_1B_ N-terminus on wild-type (α_1C_+β_2a_) and mutant (α_1C_+β_2aTM_) Ca_V_1.2 channels in the presence of Rem. * *P*<0.05 when compared to α_1C_+β_2a_ or α_1C_+β_2aTM_ using two-tailed unpaired Student's *t* test. # *P*<0.05 when compared to α_1C_+β_2a_+Rem or α_1C_+β_2aTM_+Rem using two-tailed unpaired Student's *t* test.

α_1C_NT is comprised of 153 amino acid residues. Peptide mapping (Fig. 3D) combined with co-IP (Fig. 3E) and confocal co-localization (Fig. S5) experiments suggested the Rem binding site resides in a region towards the distal end of α_1C_NT. This region is immediately upstream of transmembrane segment 1 in domain I (IS1), and shows homology (60% identical residues or conservative substitutions) among distinct Ca_V_1/Ca_V_2 α_1_-subunit isoforms (Fig. 3F). Surprisingly, despite the high sequence homology, Rem did not bind Ca_V_2.2 N-terminus (α_1B_NT) as determined either by FRET (Fig. 4A) or visual inspection of protein co-localization (not shown).

### Rem association with α_1C_NT mediates β-binding-independent inhibition of Ca_V_1.2

Does Rem binding to α_1C_NT mediate β-binding-independent Ca_V_1.2 inhibition? We addressed this question in several ways. First, given that Ca_V_2.2 α_1B_NT does not bind Rem (nor do any of the other α_1B_ intracellular domains) (Fig. 4A), we hypothesized that Ca_V_2.2 would lack a β-binding-independent form of channel inhibition. Indeed, while Rem strongly suppressed *I*_Ca_ in control cells expressing α_1B_+β_2a_ (Fig. 4B), it had no impact on α_1B_+β_2aTM_ channels (Fig. 4C). Hence, Rem inhibits Ca_V_2.2 channels solely through a β-binding-dependent mechanism. We attempted to exchange N-termini between Ca_V_1.2 α_1C_ and Ca_V_2.2 α_1B_, to determine if α_1C_NT is necessary and sufficient to reconstitute β-binding-independent Rem inhibition in Ca_V_1/Ca_V_2 channel α_1_ subunits. Unfortunately, the chimeric channels gave rise to very small currents suggesting that α_1_-subunit N-termini may have a customized, non-transferable role in the structural and/or functional maturation of individual Ca_V_1/Ca_V_2 channels.

As an alternative approach towards evaluating the functional importance of Rem/a_1C_NT association, we determined the impact of over-expressing α_1C_NT on Rem inhibition of α_1C_+β_2a_ and α_1C_+β_2aTM_ channels, respectively. We reasoned that if Rem/α_1C_NT interaction is functionally relevant then over-expressing α_1C_NT would, via competition, partially rescue Rem inhibition of α_1C_+β_2a_ channels, while fully overcoming Rem inhibition of α_1C_+β_2aTM_ channels (Fig. 4D). Indeed, these predictions were borne out in functional experiments. Over-expressing α_1C_NT partially relieved Rem inhibition of wild type Ca_V_1.2 channels (Fig. 4E; *I*_peak,0mV_ = 20.9±5.4 pA/pF, *n* = 6 for cells expressing α_1C_+β_2a_+Rem+α_1C_NT compared to *I*_peak,0mV_ = 2.8±1.2 pA/pF, *n* = 5 for α_1C_+β_2a_+Rem, *P*<0.05, Student's *t* test), while fully rescuing mutant channel currents (Fig. 4E; *I*_peak,0mV_ = 80.1±23.5 pA/pF, *n* = 8 for cells expressing α_1C_+β_2aTM_+Rem+α_1C_NT compared to *I*_peak,0 mV_ = 92.4±15.5 pA/pF, *n* = 8 for cells α_1C_+β_2aTM_). As a control experiment, α_1B_NT had no impact on Rem inhibition of mutant channels (Fig. 4E; *I*_peak,0 mV_ = 18.2±4.6 pA/pF, *n* = 5 for cells expressing α_1C_+β_2aTM_+Rem+α_1B_NT compared to *I*_peak,0 mV_ = 22.2±5.3 pA/pF, *n* = 10 for α_1C_+β_2aTM_+Rem). These results are consistent with the idea that Rem/α_1C_NT association mediates β-binding-independent Rem inhibition of Ca_V_1.2 channels.

### Distinct RGK GTPases differentially use α_1_- and β-binding dependent mechanisms to inhibit Ca_V_1.2 channels

**Figure 5:**
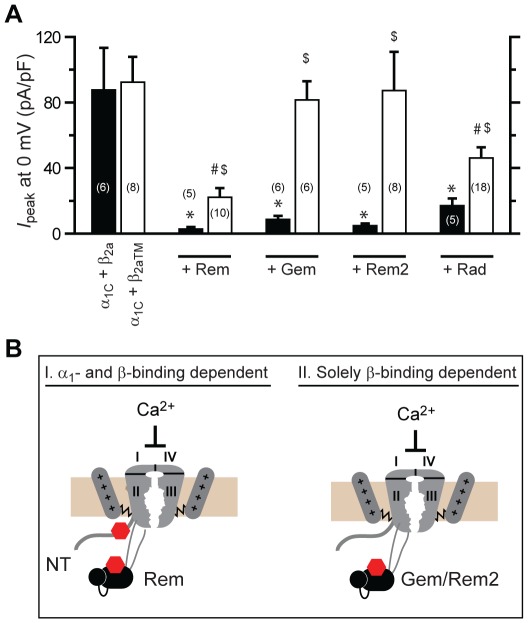
Distinct RGKs differentially use β-binding-dependent and independent mechanisms to inhibit Ca_V_1.2 channels. (A) Histogram showing impact of individual RGKs on wild-type (α_1C_+β_2a_) and mutant (α_1C_+β_2aTM_) Ca_V_1.2 channels. *, #, $ *P*<0.05 when compared to α_1C_+β_2a_, α_1C_+β_2aTM_, or α_1C_+β_2a_+RGK, respectively, using two-tailed unpaired Student's *t* test. (B) Cartoon showing dichotomy in the determinants used by distinct RGKs to inhibit Ca_V_1.2 channels.

We next examined whether the use of both α_1_- and β-binding mechanisms to inhibit Ca_V_1.2 channels is a conserved feature among the four distinct RGK GTPases. Initial indications of fundamental differences were immediately apparent from visual confocal co-localization images and co-immunoprecipitation experiments which demonstrated that unlike Rem, none of the other RGK proteins— Gem, Rem2, and Rad— bound α_1C_NT (Fig. S6). We assessed the impact of individual RGKs on either α_1C_+β_2a_ or α_1C_+β_2aTM_ channels reconstituted in HEK 293 cells, and observed a sharp dichotomy in functional responses (Fig. 5A). Whereas, all RGKs markedly inhibited *I*_Ca,L_ through wild-type α_1C_+β_2a_ channels only Rem and Rad also inhibited α_1C_+β_2aTM_ channels. Mutant α_1C_+β_2aTM_ channels were completely refractory to Gem and Rem2, explicitly demonstrating that these RGK proteins utilize only β-binding-dependent mechanisms to inhibit *I*_Ca,L_ (Fig. 5 A and B). The finding that Rad displayed both a β-binding-dependent and a β-binding-independent mode of inhibition (albeit to a lesser extent than observed for Rem) was surprising given its apparent lack of binding to α_1C_ N-terminus (Fig. S6). We speculated that Rad may bind to another intracellular domain of α_1C_ to initiate β-binding-independent inhibition of Ca_V_1.2. However, we could not detect any evidence of Rad binding to any of the other major intracellular domains of α_1C_ (Fig. S7). One possibility is that Rad may bind to α_1C_ using multiple weak interactions rather than a dominant strong binding site as we have found for Rem.

## Discussion

Amongst the myriad forms of physiological modulation of Ca_V_ channels by intracellular signaling molecules, inhibition of Ca_V_1/Ca_V_2 channels by RGKs stands out for its potency (often virtual elimination of *I*_Ca_) and indiscrimination (affects all Ca_V_1/Ca_V_2 isoforms). In this regard, RGKs behave as polar opposites to Ca_V_ channel auxiliary β subunits which interact promiscuously with all Ca_V_1/Ca_V_2 to stimulate *I*_Ca_ by increasing channel membrane trafficking and increasing single-channel open probability (*P*_o_). Given this fact, the discovery that RGKs bind βs led to the widely-held assumption that RGK/β interaction was fundamental to the mechanism of channel inhibition [15], [30]. Early renditions of this idea suggested that RGKs bound to βs and prevented their interaction with α_1_ subunits, thereby compromising channel trafficking to the membrane [10], [31], [32], and leaving channels at the cell surface in a low-*P*_o_ ‘α_1_-alone’ mode [33]. However, it was subsequently shown that RGKs do not disrupt the α_1_-β interaction leading to revised models invoking a ternary α_1_/β/RGK complex in which βs bridge α_1_ subunits and RGKs to initiate *I*_Ca_ inhibition [11], [16], [20], [34], [35]. Recently, the primacy of the RGK/β interaction in the mechanism of *I*_Ca_ inhibition has been challenged based on the interesting finding that preventing Gem interaction with β did not impair its ability to block Ca_V_2.1 (P/Q) channels [21]. In the wake of this report, it is unclear whether the RGK/β interaction has any role in the mechanism of *I*_Ca_ inhibition, or merely represents an unrelated epiphenomenon. We have investigated this issue using a β_2a_-subunit mutant that selectively loses binding to RGK proteins. The new findings presented in this work are: (1) Rem inhibits Ca_V_1.2 channels using both β-binding-dependent and β-binding–independent mechanisms; (2) binding to β is required for Rem-mediated decrease in Ca_V_1.2 channel surface density (*N*) and open probability (*P*_o_), but not *Q*_max_; (3) Rem associates with α_1C_ N-terminus to initiate β-binding-independent inhibition; (4) Rem inhibits Ca_V_2.2 channels using a solely β-binding-dependent mechanism; (5) distinct RGKs differentially use β-binding-dependent and α_1_-binding-dependent mechanisms to inhibit Ca_V_1/Ca_V_2 channels.

The finding that all four RGKs use (at least partially) β-binding-dependent mechanisms to suppress Ca_V_1.2 channels, reasserts the importance of the RGK/β interaction for *I*_Ca_ inhibition. Indeed, for Gem and Rem2, a β-binding-dependent mechanism was the sole mode for inhibiting Ca_V_1.2 channels. Similarly, Rem inhibited Ca_V_2.2 channels solely through a β-binding-dependent mechanism, indicating this phenomenon is not limited to just Ca_V_1.2 channels. Beyond β-binding-dependent inhibition, Rem and Rad also blocked Ca_V_1.2 channels in a β-binding-independent manner. For Rem, this response was mediated through an association with α_1C_NT. The discovery of an α_1C_-binding-dependent mode of RGK inhibition in Ca_V_1.2 channels aligns with the finding that Gem inhibits Ca_V_2.1 channels in a β-binding-independent (and presumably α_1A_-binding-dependent) manner [21]. Taken together with previous studies [21], [22], our data suggests a dualistic view for RGK regulation of Ca_V_1.2 channels. First, all RGKs can inhibit Ca_V_1/Ca_V_2 channels by interacting with β subunits. The essential role of βs in the functional maturation of all Ca_V_1/Ca_V_2 channels may, therefore, explain the indiscriminate nature of RGK inhibition of *I*_Ca_ through HVA Ca_V_ channels. Second, distinct RGKs can selectively inhibit specific Ca_V_1/Ca_V_2 channel isoforms by differentially binding to individual α_1_ subunits. This insight may be potentially exploited to engineer RGKs with sole selectivity for individual α_1_ subunits as a means of creating custom, isoform-specific genetically encoded Ca_V_1/Ca_V_2 channel inhibitors [17]. For Rem inhibition of Ca_V_1.2, the α_1C_-binding-dependent and β-binding-dependent mechanisms appear to be equally potent in blocking *I*_Ca,L_.

How does binding of RGK proteins to either β or α_1_ subunits actually suppress *I*_Ca_? Rem inhibition of recombinant Ca_V_1.2 channels occurs via multiple mechanisms including: decreased *N* (due to enhanced dynamin-dependent endocytosis), *P*_o_, and *Q*_max_ (due to voltage sensor immobilization) [16]. Interestingly, Rem-induced decrease in *N* and *P*_o_ (but not *Q*_max_) was β-binding-dependent. Understanding precisely how the Rem/β interaction leads to channel endocytosis and decreased *P*_o_ is an interesting question for future experiments. It is tempting to speculate that Rem-induced reduction in *Q*_max_ (voltage sensor immobilization) underlies α_1C_-binding-dependent inhibition of Ca_V_1.2. Nevertheless, we cannot rule out that Rem binding to α_1C_NT may also inhibit channel *P*_o_ using a parallel mechanism that is independent of voltage sensor immobilization. Such mechanistic details may potentially be resolved by evaluating the structural determinants on Rem necessary for α_1C_-binding-dependent inhibition [16].

Over the last decade, several groups have investigated mechanisms of RGK GTPase inhibition of Ca_V_ channels, sometimes with discrepant results [10], [11], [12], [16], [21], [31], [35], [36], [37]. Often, across the various groups, these studies have involved different RGKs and distinct Ca_V_1/Ca_V_2 channel types, as well as varied experimental systems. This work produces the new insight that the mode of RGK-mediated Ca_V_ channel inhibition is customized at both the channel and GTPase level. Hence, a particular RGK can employ divergent mechanisms to block distinct Ca_V_ channel types, while a specific Ca_V_ channel isoform can be inhibited by different RGKs with diverse mechanisms. This perspective may help explain some of the inconsistent results previously published regarding RGK regulation of Ca_V_ channels.

In conclusion, this work contributes to the growing realization that the seemingly simple phenomenon of RGK inhibition of Ca_V_1/Ca_V_2 channels is underlain by a rich variety of mechanisms and structural determinants [16], [36]. Such mechanistic complexity may be physiologically relevant as it could significantly enrich the functional versatility of RGKs as Ca^2+^ channel blockers in excitable cells. For example, RGK inhibition of *I*_Ca_ could occur on different timescales depending on the mode of block of Ca_V_ channels– β-binding-dependent decreases in *N* could lead to long-term reductions in current, while β-binding-independent regulation of *Q*_max_ produces short-term tuning of *I*_Ca_. In-depth understanding of the complexities underlying RGK regulation of *I*_Ca_ will be important for deciphering such physiological dimensions of this channel modulation, and may be potentially exploited to create custom genetically encoded Ca_V_ channel blockers for specific applications.

## Materials and Methods

### cDNA cloning

XFP-tagged RGK constructs [mouse Rem (NM_009047); human Gem (NM_181702); human Rem2 (NM_173527); mouse Rad (NM_019662)] were generated by first polymerase chain reaction (PCR) amplifying and cloning XFP into pcDNA4.1 (Invitrogen) using *Kpn*I and *BamH*I sites. Subsequently, RGK constructs were PCR amplified and cloned downstream of XFP using *BamH*I and *EcoR*I sites. To generate CFP-Rem_265_-C1_PKCγ_, we used overlap extension PCR to fuse residues 26–89 of mouse PKCγ [38] to the C terminus of Rem_265_. The fusion product was subsequently cloned downstream of CFP using *BamH*I and *EcoR*I sites. CFP-α_1C_ intracellular loops constructs were amplified by PCR and cloned downstream of the XFP molecule using *BamH*I and *EcoR*I sites. To generate XFP-tagged Ca_V_β constructs, we PCR amplified and cloned XFP into pAd CMV using *BamH*I and *Xba*I sites. Ca_V_βs were amplified by PCR and cloned upstream of the XFP molecule using *Nhe*I and *BamH*I sites. Point mutations in β were generated using QuikChange Site-Directed Mutagenesis Kit (Stratagene). The thirteen-residue bungarotoxin binding site [BBS] [39] was engineered into the domain II S5–S6 extracellular loop of α_1C_ at residue 713 using unique restriction enzyme sites, *Stu*I and *BbrP*I. Primers that extended from the unique restriction sites were used together with primers containing the BBS sequence in an overlap extension PCR reaction. The overlap extension product was directly ligated into α_1C_-YFP to generate α_1C_[BBS]-YFP.

### All PCR products were verified by sequencing

#### Cell culture and transfection

Low-passage-number HEK 293 cells (gift from Dr. Robert Kass, Columbia University) [40] were maintained in DMEM supplemented with 10% FBS and 100 µg ml^−1^ penicillin-streptomycin. HEK 293 cells cultured in 6-cm tissue culture dishes were transiently transfected with Ca_V_1.2α_1C_ (6 µg), β_2a_ (6 µg), T antigen (2 µg), and the appropriate RGK construct (4 µg), using the calcium phosphate precipitation method. Cells were washed with PBS 5–8 h after transfection and maintained in supplemented DMEM. For confocal microscopy experiments, transfected HEK 293 cells were replated onto fibronectin-coated culture dishes with No. 0 glass coverslip bottoms (MaTek). For electrophysiology experiments cells were replated onto fibronectin-coated glass coverslips 24 h after transfection.

### Electrophysiology

Whole-cell recordings were conducted 48–72 h after transfection using an EPC-8 or EPC-10 patch clamp amplifier (HEKA Electronics) controlled by PULSE software (HEKA). Micropipettes were fashioned from 1.5-mm thin-walled glass with filament (WPI Instruments), and filled with internal solution containing (in mM): 135 cesium methanesulphonate (MeSO_3_), 5 CsCl, 5 EGTA, 1 MgCl_2_, 4 MgATP (added fresh) and 10 HEPES (pH 7.3). Series resistance was typically 1.5–2 MΩ. There was no electronic series resistance compensation. External solution contained (in mM): 140 tetraethylammonium-MeSO_3_, 5 BaCl_2_, and 10 HEPES (pH 7.3). Whole-cell *I*–*V* curves were generated from a family of step depolarizations (−40 to +100 mV from a holding potential of −90 mV). Currents were sampled at 25 kHz and filtered at 5 or 10 kHz. Traces were acquired at a repetition interval of 6 s. Leak and capacitive currents were subtracted using a P/8 protocol.

### Labeling of cell surface Ca_V_1.2 channels with QD_655_

Transfected cells were washed twice with PBS containing calcium and magnesium (pH 7.4, 0.9 mM CaCl_2_ and 0.49 mM MgCl_2_), and incubated with 1 µM biotinylated α-bungarotoxin in DMEM/3% BSA in the dark for 1 h at room temperature. Cells were washed twice with DMEM/3% BSA, and incubated with 10 nM streptavidin-conjugated QD_655_ for 1 h at 4°C in the dark. For confocal microscopy, cells were washed with PBS, and imaged in the same buffer. For flow cytometry, cells were harvested with trypsin, washed with PBS and assayed in the same buffer.

### Confocal microscopy

Static images of α_1C_[BBS]-YFP, XFP-Rem constructs and quantum dots signal were observed using a Leica TCS SPL AOBS MP Confocal microscope system and a 40× oil objective (HCX PL APO 1.25-.75 NA). HEK 293 cells expressing CFP/YFP fusion proteins were imaged using a 458/514-nm Argon laser line for excitation and red signals were imaged using a 633-nm helium-neon laser line for excitation.

### Flow cytometry

Cells were counted using a BD LSRII Cell Analyzer. HEK 293 cells expressing CFP/YFP fusion proteins were excited at 407 and 488-nm, respectively, and red signal was excited at 633-nm. For each group of experiments we used isochronal untransfected and single color controls to manually set the appropriate gain settings for each fluorophore to ensure signals remained in the linear range and to set threshold values. The same gain settings were then used for assaying all isochronal transfection samples. Flow cytometry data were analyzed using FlowJo software.

### Immunoprecipitation and immunoblotting

Confluent cultures of HEK 293 cells plated in 6-cm tissue culture dishes were harvested 48 h after transfection. Cells were washed in PBS and resuspended in 0.5 mL cold lysis buffer (50 mmol/L Tris-HCl, 150 mmol/L NaCl, 1% NP-40) containing 1× protease inhibitor cocktail for 30 minutes. Cell lysates were centrifuged at 10,000×g for 15 minutes at 4°C, and the supernatant precleared by incubation with 50 µL protein G beads slurry for 1 h. The mixture was centrifuged and the resulting supernatant incubated with 4 µg primary antibody [Santa Cruz Biotechnology: anti-Rem (SC58472); anti-Gem (SC19753); anti-Rem2 (SC160720); anti-Rad (SC49714)] and 50 µL protein G slurry for 1 h on a rotator. The mixture was again centrifuged, and the pellet washed four times with lysis buffer. 50 µL Laemmli sample buffer was added to the bead pellet and the mixture vortexed and heated (90°–100°C for 10 minutes). The sample was centrifuged and the supernatant loaded onto a gel for subsequent SDS-PAGE and Western blot analyses. For immunoblots, primary antibodies to GFP (Invitrogen, A6455) were detected by horseradish peroxidase-conjugated secondary antibodies (goat-anti rabbit obtained from Thermo Scientific, 32260) and enhanced chemiluminescence.

### Fluorescence resonance energy transfer (FRET)

Determination of RGK-α_1_ subunit intracellular domain interactions in live cells was accomplished using the three-cube FRET algorithm as previously described [27], [28]. Cells transfected with XFP-tagged proteins were washed with Tyrode's solution and placed on an inverted microscope equipped for epifluorescence. Individual cells were excited using a 150-W Xenon arc lamp light source, and epifluorescence emission signals measured with a photomultiplier tube were integrated by a fluorometer and digitized. For each cell, three successive measurements were taken with filter cube sets optimum for measuring CFP, YFP, and FRET signals, respectively. Background and autofluorescence levels were determined by averages from single untransfected cells, and subtracted from experimental values from each cube. The FRET ratio (*FR*) was calculated from background-corrected experimental measurements as previously described [27], [28].

### Data and statistical analyses

Data were analyzed off-line using PulseFit (HEKA), Microsoft Excel and Origin software. Statistical analyses were performed in Origin using built-in functions. Statistically significant differences between means (*P*<0.05) were determined using two-tailed unpaired Student's *t* test. Data are presented as means ± S.E.M.

## Supporting Information

Figure S1 **Evidence that β_TM_ loses binding to Rem.** (A) Confocal images of a HEK 293 cell co-expressing CFP-Rem_265_-C1_PKC_ and wild type YFP-β_3_. Under basal conditions both CFP and YFP fluorescence are diffusely distributed in the cytosol. Upon addition of 1 µM PdBu (5 min), CFP-Rem_265_-C1_PKC_ is recruited to the nuclear and plasma membrane. The sub-cellular localization of YFP-β_3_ dynamically follows that of CFP-Rem_265_-C1_PKC_, providing visual evidence of an interaction between the two proteins. Scale bar, 5 µm. (B) A mutant β_3_ featuring three point mutations, YFP-β_TM_, does not bind CFP-Rem_265_-C1_PKC_, as reported by the dynamic sub-cellular co-localization assay. (C) Co-immunoprecipitation assay indicates YFP-β_2a_ associates with CFP-Rem, and that this interaction is lost with YFP-β_2aTM_.
(TIF)

Figure S2 **Exemplar raw data from flow cytometry experiments used to determine the relative surface density of Ca_V_1.2 channels.** (A) Confocal images showing quantum dot labeling of cells transfected with α_1C_[BBS]-YFP+β_2a_ ± CFP-Rem (*left*) and α_1C_[BBS]-YFP+β_2aTM_ ± CFP-Rem (*right*). Images are reproduced from Fig. 2A, B. Scale bar, 25 µm. (B) Raw data from isochronal flow cytometry experiments showing fluorescence intensity of QD_655_ versus YFP signals for cells expressing α_1C_[BBS]-YFP+β_2a_+CFP-Rem (*left*) and α_1C_[BBS]-YFP+β_2aTM_+CFP-Rem (*right*). 50,000 cells were counted for each condition. Vertical and horizontal lines are threshold values set based on isochronal experiments using untransfected and single color control cells. Each dot represents a single cell. Dots have been arbitrarily color coded to facilitate visualization of distinct populations. Loosely, green dots represent α_1C_[BBS]-YFP-positive cells that lack appreciable trafficking to the membrane (low QD_655_ signal), while red dots represent α_1C_[BBS]-YFP-positive cells that display robust Ca_V_1.2 channel trafficking to the surface (high QD_655_ signal). Black dots in the bottom left quadrant correspond to untransfected cells.
(TIF)

Figure S3 **Histogram showing estimates of donor∶acceptor ratio (** ***N*** **_D_/** ***N*** **_A_) for FRET experiments shown in** **Fig. 3** **.**
(TIF)

Figure S4 **Visual evidence that Rem selectively binds α_1C_ N-terminus.** (A) Representative confocal images showing sub-cellular localization of YFP-tagged α_1C_ intracellular domains when expressed alone in HEK 293 cells. Aside from I–II loop, which autonomously targets to the membrane and nucleus, all other α_1C_ intracellular domains show mostly diffuse distribution throughout the cell. Scale bar, 5 µm. (B) *Top row*, representative images of YFP-Rem demonstrate that this protein is membrane enriched when expressed in HEK 293 cells. *Bottom row*, representative images showing sub-cellular localization of CFP-tagged α_1C_ intracellular loops co-expressed with YFP-Rem. Only CFP-α_1C_NT demonstrated redistribution from the cytosol to the plasma membrane when co-expressed with YFP-Rem. (C) Line scan analyses of CFP fluorescence from cells co-expressing YFP-Rem and CFP-tagged α_1C_ intracellular loops. Membrane localization of CFP-α_1C_NT and CFP-α_1C_I–II is evident from the sharp twin peaks of fluorescent signal separated by (cytoplasmic) regions with lower fluorescence intensity. Line scans were drawn to avoid the nucleus and areas with clustered fluorescence. (D) Relative membrane to cytosol fluorescence intensity ratios for CFP-tagged α_1C_ intracellular domains either expressed alone or together with YFP-Rem in HEK 293 cells. Absence of membrane targeting results in a ratio of one, while membrane localization/enrichment of a protein yields a ratio greater than one. By this analysis, only CFP-α_1C_NT showed an increase in membrane localization when co-expressed with YFP-Rem. CFP-α_1C_I–II showed a relative decrement in membrane localization when co-expressed with YFP-Rem, perhaps reflecting a competition for membrane binding sites.
(TIF)

Figure S5 **Mapping the Rem binding site in α_1C_ N-terminus.** (A) Schematic of α_1C_NT peptide fragments used to map Rem binding site. (B) Co-localization pattern of specific YFP-tagged α_1C_ N-terminus fragments with CFP-Rem at the plasma membrane suggests Rem binds the distal end of α_1C_ N-terminus. Scale bar, 5 µm. (C) Relative membrane to cytosol fluorescence intensity ratios for YFP-tagged α_1C_NT fragments co-expressed with CFP-Rem. Ratios greater than unity indicate membrane targeting/enrichment of fluorescence signal. Line scan analyses avoided the nucleus and clustered fluorescence signals from cytosolic areas.
(TIF)

Figure S6 **Lack of interaction of Gem, Rem2, and Rad with α_1C_ N-terminus.** (A) Confocal images of YFP-α_1C_NT with CFP-tagged Gem, Rem2, and Rad show little co-localization. Scale bar, 5 µm. (B) Relative membrane to cytosol fluorescence intensity ratios for YFP-α_1C_NT co-expressed with distinct CFP-tagged RGK proteins. (C) Co-immuoprecipitation assay to probe for α_1C_NT interaction with Gem, Rem2, or Rad provides no evidence of an association.
(TIF)

Figure S7 **Lack of interaction of Rad with α_1C_ intracellular loops.** (A) Confocal images of mCherry-Rad and CFP-tagged α_1C_ intracellular loops and termini show no evidence of co-localization. Scale bar, 5 µm. (B) Relative membrane to cytosol fluorescence intensity ratios for YFP-tagged α_1C_ intracellular loops co-expressed with distinct mCherry-tagged Rad. (C) Co-immunoprecipitation assays indicate no interaction between Rad and the major α_1C_ intracellular loops.
(TIF)
