# Supplementary figures and images for: Distinct RGK GTPases Differentially Use α1- and Auxiliary β-Binding-Dependent Mechanisms to Inhibit CaV1.2/CaV2.2 Channels
Source: PLoS One. 2012 May 10;7(5):e37079. doi: 10.1371/journal.pone.0037079 (PMC3349659; doi:10.1371/journal.pone.0037079)

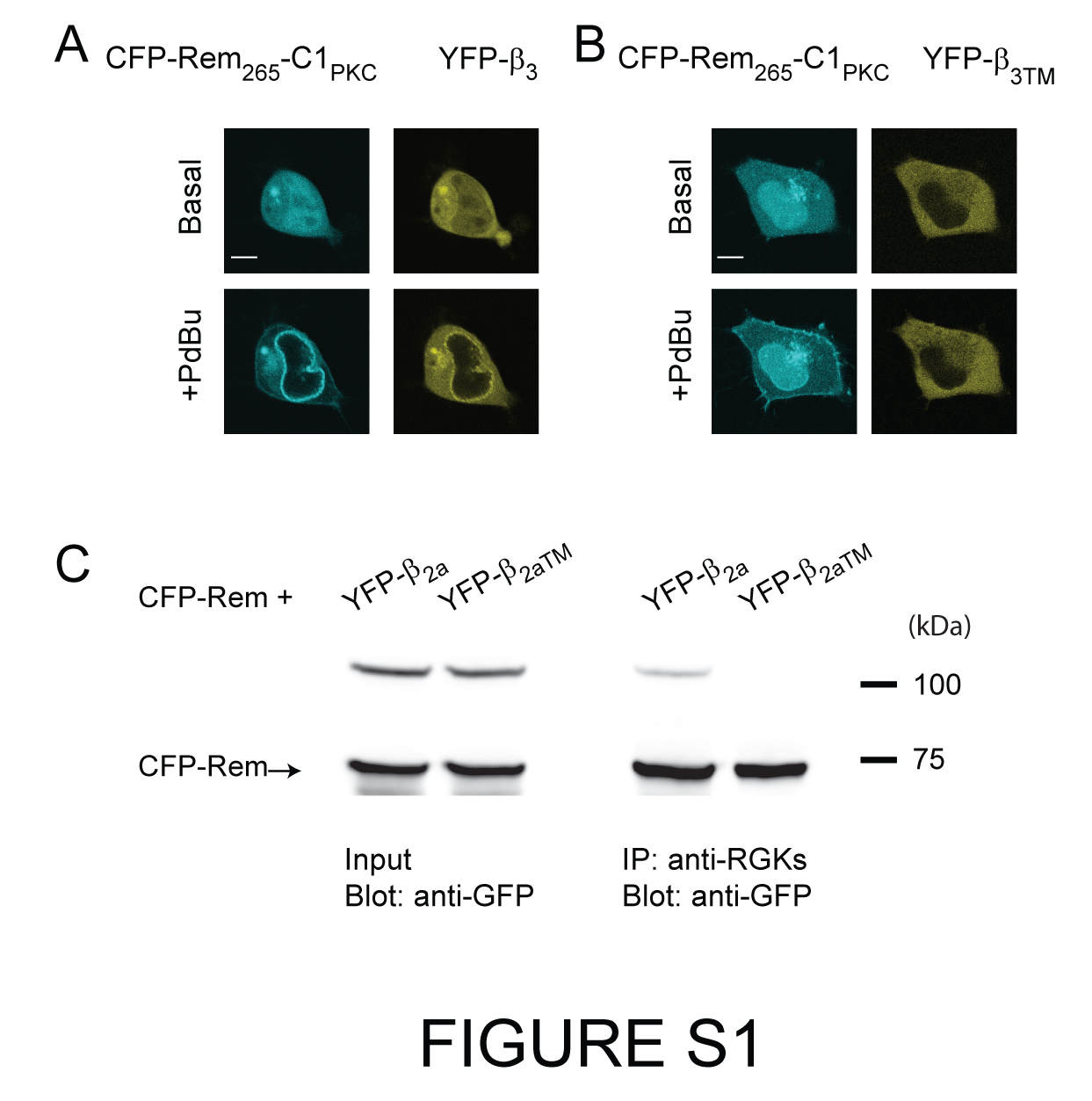

Supplement: Figure S1 — Evidence that βTM loses binding to Rem. (A) Confocal images of a HEK 293 cell co-expressing CFP-Rem265-C1PKC and wild type YFP-β3. Under basal conditions both CFP and YFP fluorescence are diffusely distributed in the cytosol. Upon addition of 1 µM PdBu (5 min), CFP-Rem265-C1PKC is recruited to the nuclear and plasma membrane. The sub-cellular localization of YFP-β3 dynamically follows that of CFP-Rem265-C1PKC, providing visual evidence of an interaction between the two proteins. Scale bar, 5 µm. (B) A mutant β3 featuring three point mutations, YFP-βTM, does not bind CFP-Rem265-C1PKC, as reported by the dynamic sub-cellular co-localization assay. (C) Co-immunoprecipitation assay indicates YFP-β2a associates with CFP-Rem, and that this interaction is lost with YFP-β2aTM. (TIF) [file pone.0037079.s001.tif]

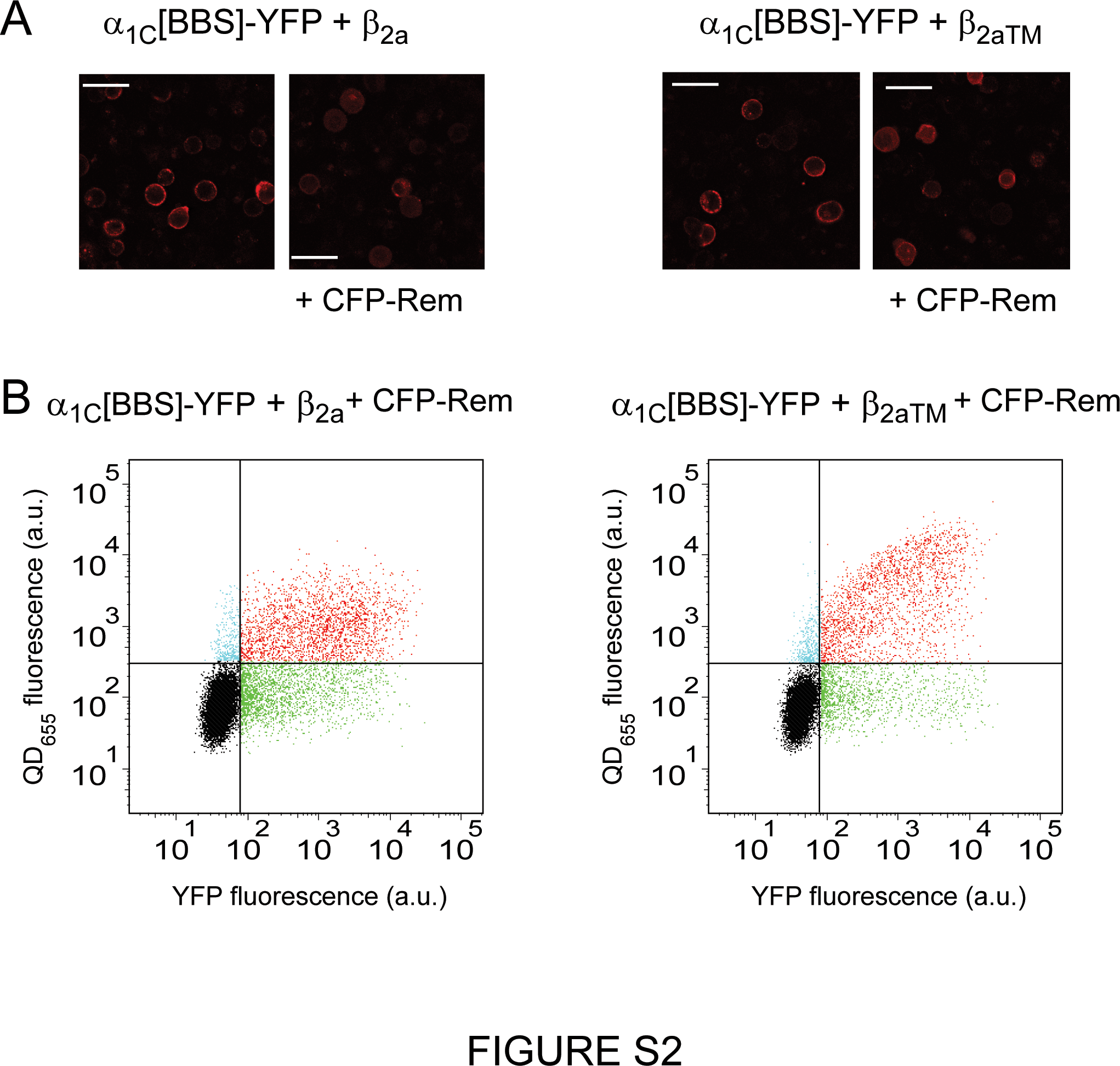

Supplement: Figure S2 — Exemplar raw data from flow cytometry experiments used to determine the relative surface density of CaV1.2 channels. (A) Confocal images showing quantum dot labeling of cells transfected with α1C[BBS]-YFP+β2a ± CFP-Rem (left) and α1C[BBS]-YFP+β2aTM ± CFP-Rem (right). Images are reproduced from Fig. 2A, B. Scale bar, 25 µm. (B) Raw data from isochronal flow cytometry experiments showing fluorescence intensity of QD655 versus YFP signals for cells expressing α1C[BBS]-YFP+β2a+CFP-Rem (left) and α1C[BBS]-YFP+β2aTM+CFP-Rem (right). 50,000 cells were counted for each condition. Vertical and horizontal lines are threshold values set based on isochronal experiments using untransfected and single color control cells. Each dot represents a single cell. Dots have been arbitrarily color coded to facilitate visualization of distinct populations. Loosely, green dots represent α1C[BBS]-YFP-positive cells that lack appreciable trafficking to the membrane (low QD655 signal), while red dots represent α1C[BBS]-YFP-positive cells that display robust CaV1.2 channel trafficking to the surface (high QD655 signal). Black dots in the bottom left quadrant correspond to untransfected cells. (TIF) [file pone.0037079.s002.tif]

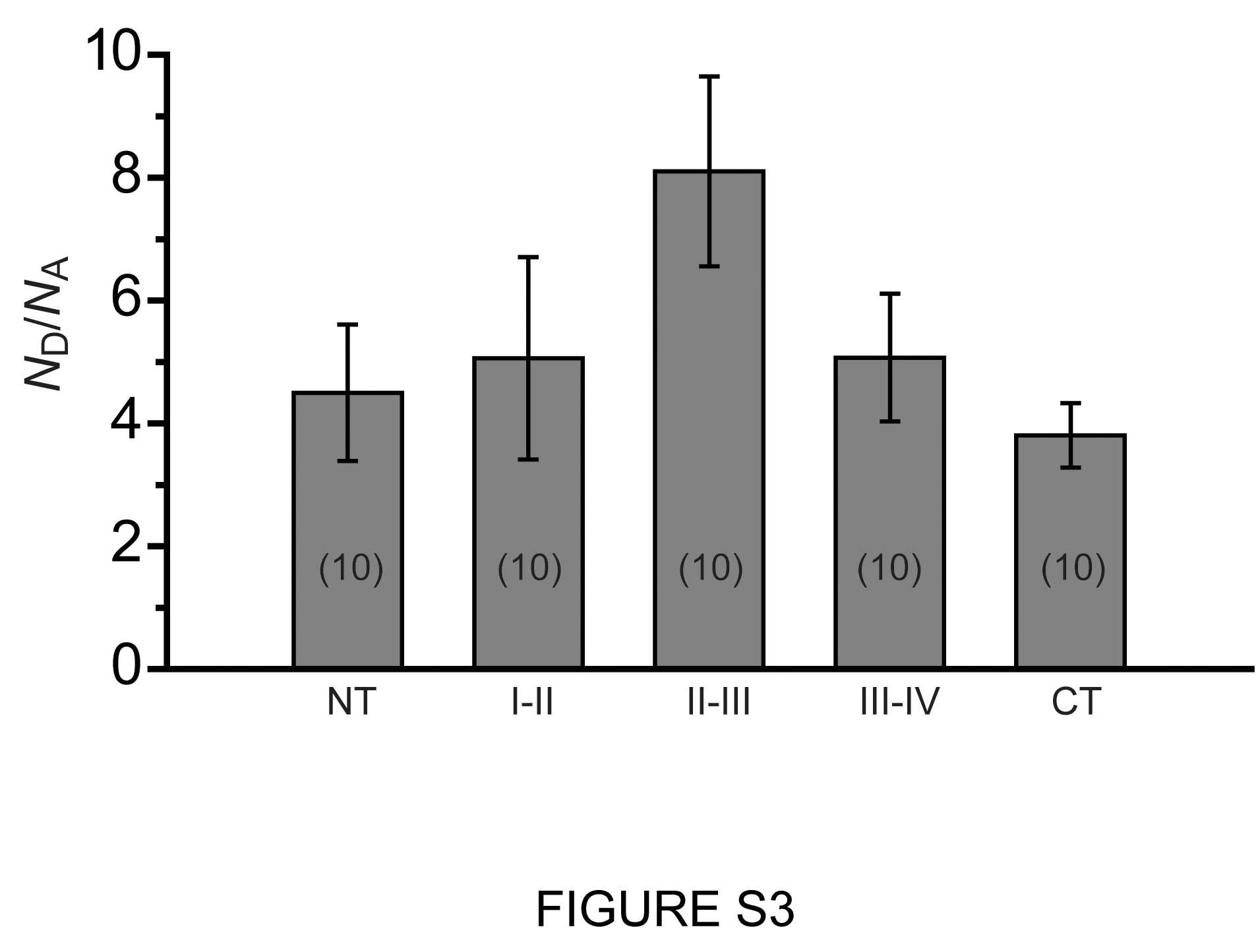

Supplement: Figure S3 — Histogram showing estimates of donor∶acceptor ratio ( N D/ N A) for FRET experiments shown in Fig. 3 . (TIF) [file pone.0037079.s003.tif]

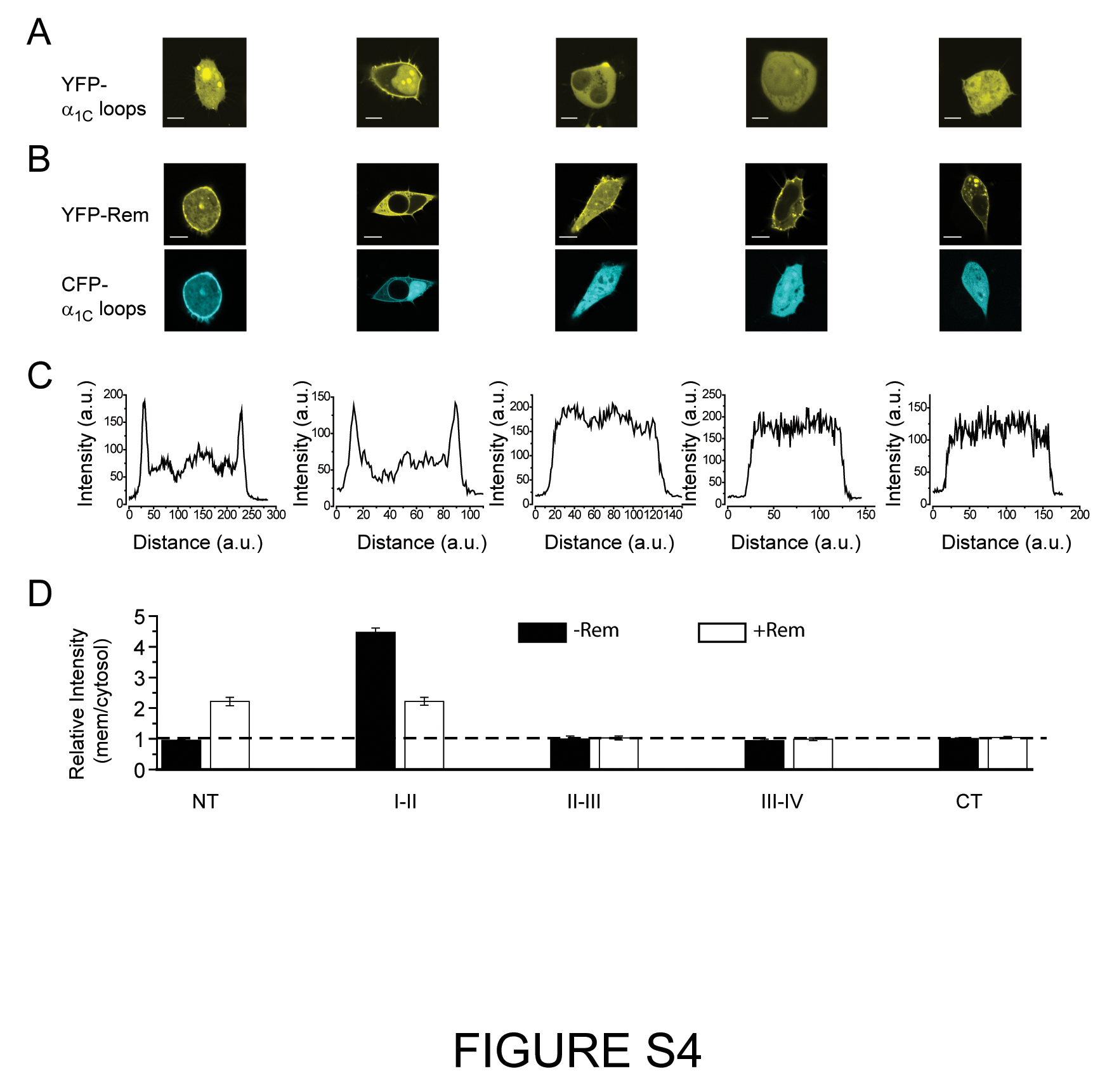

Supplement: Figure S4 — Visual evidence that Rem selectively binds α1C N-terminus. (A) Representative confocal images showing sub-cellular localization of YFP-tagged α1C intracellular domains when expressed alone in HEK 293 cells. Aside from I–II loop, which autonomously targets to the membrane and nucleus, all other α1C intracellular domains show mostly diffuse distribution throughout the cell. Scale bar, 5 µm. (B) Top row, representative images of YFP-Rem demonstrate that this protein is membrane enriched when expressed in HEK 293 cells. Bottom row, representative images showing sub-cellular localization of CFP-tagged α1C intracellular loops co-expressed with YFP-Rem. Only CFP-α1CNT demonstrated redistribution from the cytosol to the plasma membrane when co-expressed with YFP-Rem. (C) Line scan analyses of CFP fluorescence from cells co-expressing YFP-Rem and CFP-tagged α1C intracellular loops. Membrane localization of CFP-α1CNT and CFP-α1CI–II is evident from the sharp twin peaks of fluorescent signal separated by (cytoplasmic) regions with lower fluorescence intensity. Line scans were drawn to avoid the nucleus and areas with clustered fluorescence. (D) Relative membrane to cytosol fluorescence intensity ratios for CFP-tagged α1C intracellular domains either expressed alone or together with YFP-Rem in HEK 293 cells. Absence of membrane targeting results in a ratio of one, while membrane localization/enrichment of a protein yields a ratio greater than one. By this analysis, only CFP-α1CNT showed an increase in membrane localization when co-expressed with YFP-Rem. CFP-α1CI–II showed a relative decrement in membrane localization when co-expressed with YFP-Rem, perhaps reflecting a competition for membrane binding sites. (TIF) [file pone.0037079.s004.tif]

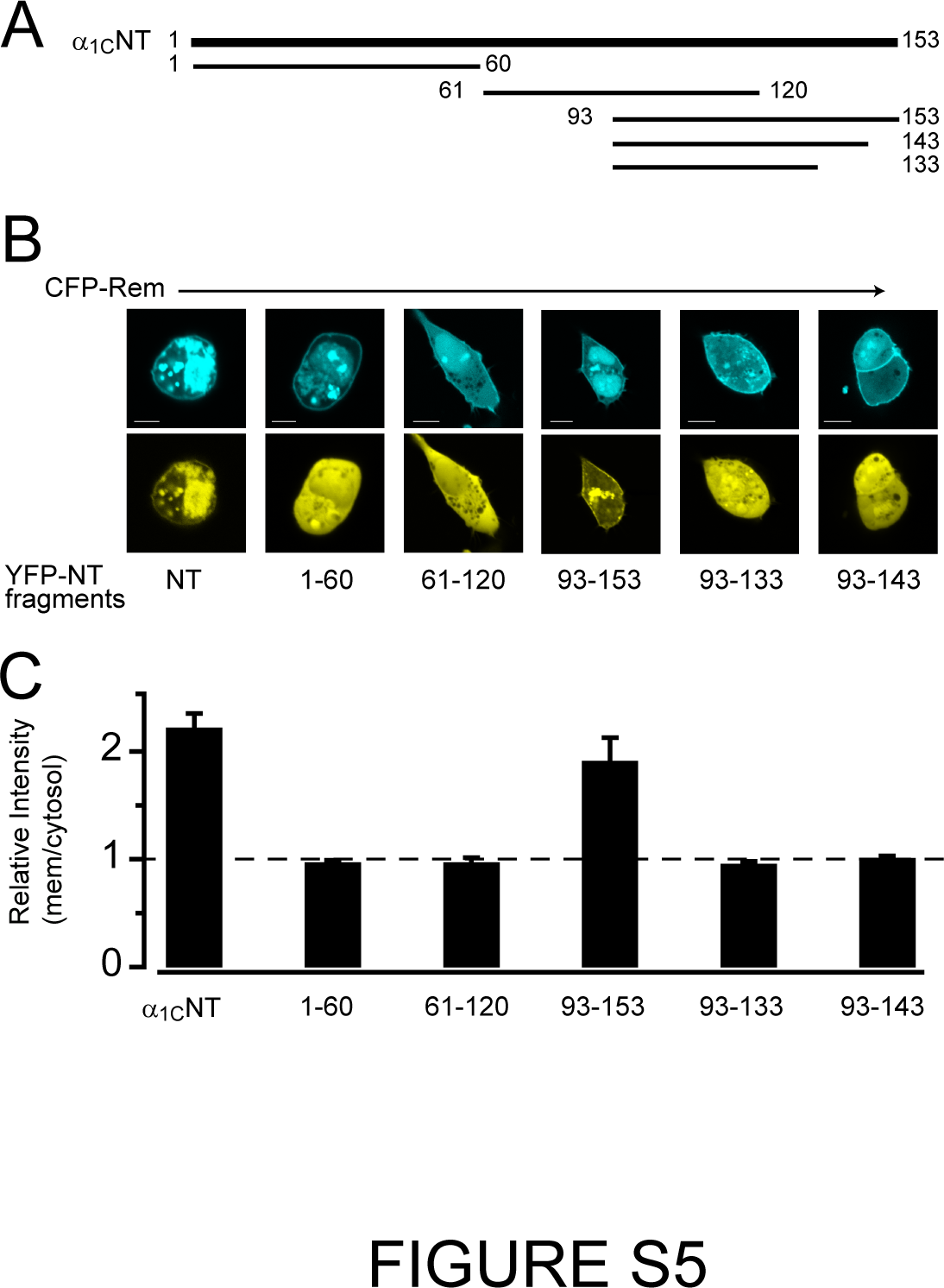

Supplement: Figure S5 — Mapping the Rem binding site in α1C N-terminus. (A) Schematic of α1CNT peptide fragments used to map Rem binding site. (B) Co-localization pattern of specific YFP-tagged α1C N-terminus fragments with CFP-Rem at the plasma membrane suggests Rem binds the distal end of α1C N-terminus. Scale bar, 5 µm. (C) Relative membrane to cytosol fluorescence intensity ratios for YFP-tagged α1CNT fragments co-expressed with CFP-Rem. Ratios greater than unity indicate membrane targeting/enrichment of fluorescence signal. Line scan analyses avoided the nucleus and clustered fluorescence signals from cytosolic areas. (TIF) [file pone.0037079.s005.tif]

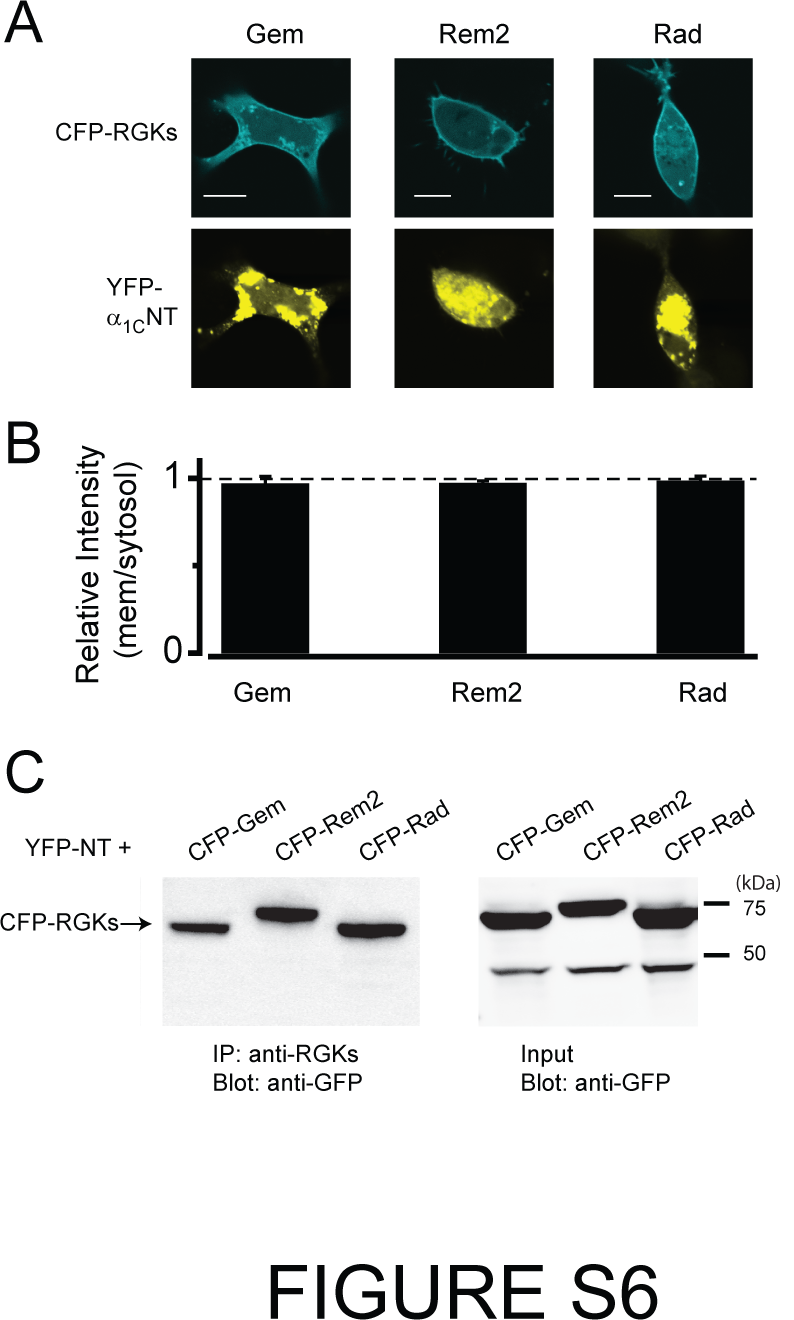

Supplement: Figure S6 — Lack of interaction of Gem, Rem2, and Rad with α1C N-terminus. (A) Confocal images of YFP-α1CNT with CFP-tagged Gem, Rem2, and Rad show little co-localization. Scale bar, 5 µm. (B) Relative membrane to cytosol fluorescence intensity ratios for YFP-α1CNT co-expressed with distinct CFP-tagged RGK proteins. (C) Co-immuoprecipitation assay to probe for α1CNT interaction with Gem, Rem2, or Rad provides no evidence of an association. (TIF) [file pone.0037079.s006.tif]

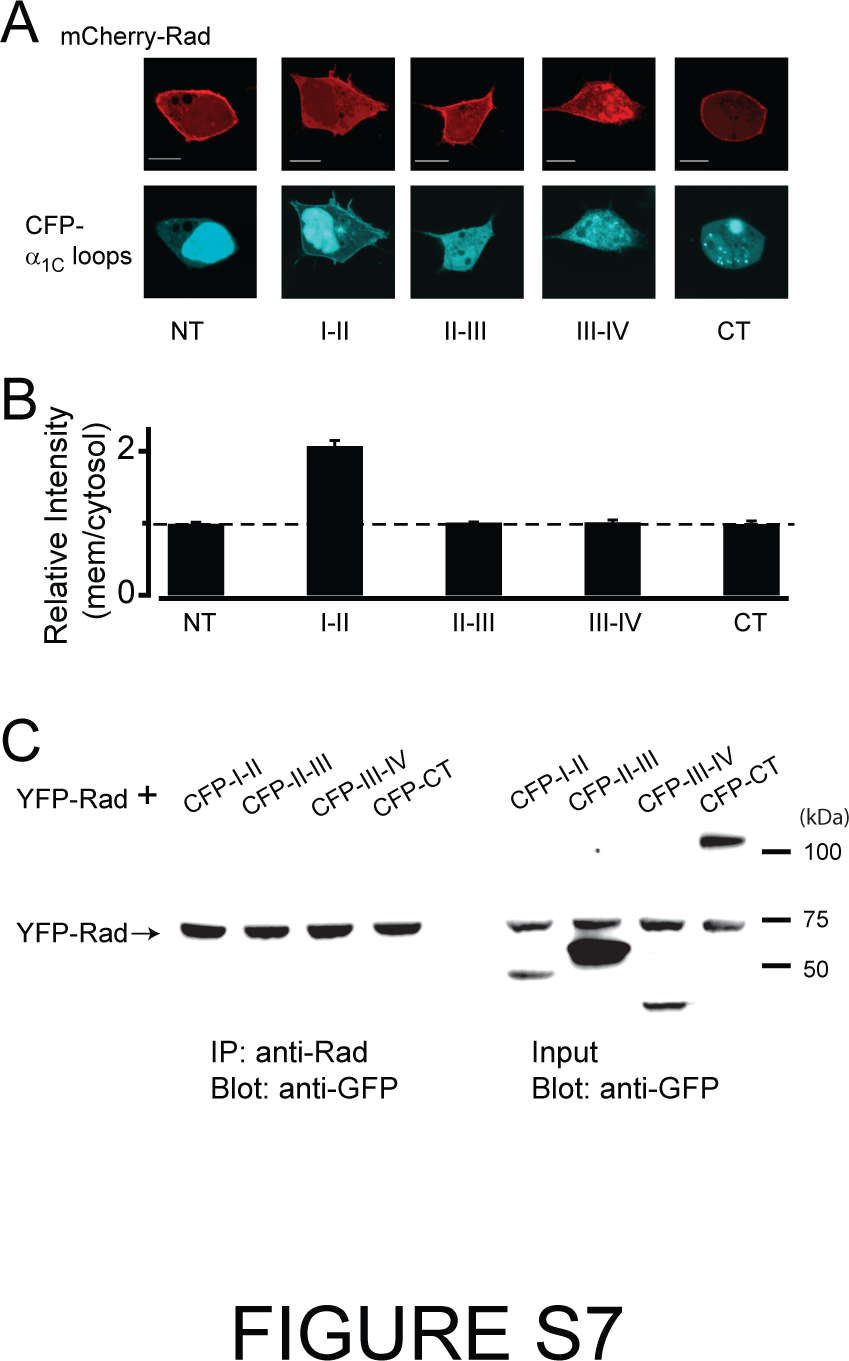

Supplement: Figure S7 — Lack of interaction of Rad with α1C intracellular loops. (A) Confocal images of mCherry-Rad and CFP-tagged α1C intracellular loops and termini show no evidence of co-localization. Scale bar, 5 µm. (B) Relative membrane to cytosol fluorescence intensity ratios for YFP-tagged α1C intracellular loops co-expressed with distinct mCherry-tagged Rad. (C) Co-immunoprecipitation assays indicate no interaction between Rad and the major α1C intracellular loops. (TIF) [file pone.0037079.s007.tif]
